# Immune-mediated inflammatory diseases and periodontal disease: a bidirectional two-sample mendelian randomization study

**DOI:** 10.1186/s12865-024-00634-y

**Published:** 2024-06-28

**Authors:** Rui Zhang, Hairong Ma, Dan Wang, Hualin Zhang

**Affiliations:** 1 Department of General Stomatology, General Hospital of Ningxia Medical University, Yinchuan, 750004 China; 2 College of Stomatology, Ningxia Medical University, Yinchuan, 750004 China; 3 Department of Stomatology, Qingtongxia Hospital of Traditional Chinese Medicine, Ningxia, 751600 China; 4 Ningxia Province Key Laboratory of Oral Diseases Research, Ningxia Medical University, Yinchuan, 750004 China

**Keywords:** Immune-mediated inflammatory diseases, Periodontal disease, Mendelian randomization, Causal effect

## Abstract

**Background:**

Previous observational studies have shown a bidirectional association between immune-mediated inflammatory disorders (IMID) and periodontal disease. However, evidence regarding the causal role of IMID and periodontal disease is still lacking. Therefore, we conducted a bidirectional two-sample Mendelian randomization (MR) study to uncover the potential genetic causal effects between IMID and periodontal disease.

**Methods:**

Bidirectional two-sample MR analysis was employed. Data for ten IMIDs were sourced from genome-wide association studies (GWAS) conducted by the FinnGen Consortium (range from 1023 to 36321 cases) and UK Biobank (UKB) (range from 150 to 17574 cases). Furthermore, GWAS data for periodontal disease were obtained from the FinnGen Consortium (87497 cases), UKB (458 cases), and Gene Lifestyle Interactions in Dental Endpoints (GLIDE) consortium (17,353 periodontitis cases). Subsequently, the causal relationships were analyzed by random effects inverse variance weighting, weighted median, and MR-Egger. Sensitivity analyses were performed using the Cochrane Q test, funnel plot, and Mr-Egger intercept test to ensure robustness. Eventually, replication analysis and meta-analysis across different databases were carried out.

**Results:**

Systemic lupus erythematosus (SLE) [IVW: OR = 1.079 (95% CI: 1.032–1.128) and *P* < 0.001], Sjogren syndrome [IVW: OR = 1.082 (95% CI: 1.012–1.157) and *P* = 0.022] and hypothyroidism [IVW: OR = 1.52 (95% CI: 1.13–2.04) and *P* = 0.005] may increase the risk of periodontal disease. In addition, periodontal disease may reduce the risk of SLE [IVW: OR = 0.8079 (95% CI: 0.6764–0.9650) and *P* = 0.019] and hyperthyroidism [IVW: OR = 5.59*10^–9^ (95% CI: 1.43*10^–15^-2.18*10^–2^) and* P* = 0.014]. Meta-analysis indicated a causal correlation between SLE and an increased risk of periodontal disease: [OR = 1.08 (95% CI: 1.03–1.13), *P* = 0.0009]. No significant evidence suggests bilateral causal relationships between other IMIDs and periodontal disease. No significant estimation of heterogeneity or pleiotropy is detected.

**Conclusions:**

Our study has confirmed a genetic causal relationship between IMIDs and periodontal disease, thereby unveiling novel potential mechanisms underlying IMIDs and periodontal disease. This discovery is promising in fostering interdisciplinary collaboration between clinicians and stomatologists to facilitate appropriate and precise screening, prevention, and early treatment of IMIDs and periodontal disease.

**Supplementary Information:**

The online version contains supplementary material available at 10.1186/s12865-024-00634-y.

## Introduction

Periodontal disease, one of the major oral diseases, encompasses a variety of diseases affecting the periodontal supporting tissue (both the soft and hard tissue around the teeth), of which the most common forms are periodontitis and gingivitis [1, 2]. In its advanced stage, periodontal disease can lead to tooth displacement and looseness, significantly impairing the patient's ability to chew and negatively impacting their quality of life. Periodontal disease is reported to affect approximately 538 million individuals worldwide [3]. Furthermore, periodontal disease witnessed a notable growth rate of 25.4% from 2005 to 2015 [4]. With an ever-aging population, the incidence of periodontal disease will inevitably escalate. Therefore, the prevention of periodontal disease is particularly essential. The etiology of periodontal disease has been demonstrated to range from complex factors, with plaque microorganisms and host immune response being the main and direct causes, while their interaction modulates the severity of the disease [5]. Several studies have revealed that the host’s immune-inflammatory reaction to bacterial colonization within the biofilm is responsible for mediating the development of periodontal disease and regulating the susceptibility of patients [6]. Additionally, there are intricate correlations between periodontal disease and numerous systemic diseases. Observational studies have demonstrated associations between periodontal disease and a variety of systemic immune disorders, such as immune-mediated inflammatory disorders (IMID) [7, 8]. In view of the fact that the immune-inflammatory response is the potential pathogenic mechanism of periodontal disease, whether immune system diseases affect periodontal disease deserves further investigation.

IMID is an encompassing designation for a group of illnesses characterized by a similar inflammatory pathway and is defined by an immune system malfunction that can induce chronic inflammation within any organ in the body [9, 10]. IMID includes Systemic lupus erythematosus (SLE), inflammatory bowel disease (IBD), and ulcerative colitis (UC), among others, all manifesting a diverse set of clinical symptoms. With a prevalence of approximately 80 instances per 100,000 person-years, IMIDs impose a negative impact on 3-5% of the population [11]. However, the pathogenesis of IMIDs is still not fully understood. Observational studies have pointed out that in susceptible individuals, dysregulation of homeostasis within periodontal local inflammation and immunity may trigger systemic immune responses through innate and adaptive mechanisms, thus inducing autoimmune diseases [12, 13]. It is imperative to improve the management of IMID and periodontal disease by identifying the potential causal relationship between these two diseases. More and more studies have focused on exploring the relationship and internal mechanisms between IMID and periodontal disease, but the specific causal relationship has not been clearly pointed out [14–16]. Moreover, these studies have certain limitations, including a restricted sample size and insufficient test efficiency. Additionally, some observational studies and systematic review analyses exhibit selection and information bias, and the control of confounding factors such as smoking and mental stress may have some bias.

In recent years, Mendelian randomization (MR) analysis has gained significant traction, primarily for determining causality between exposure variables and outcomes [17]. MR, in particular, employs single nucleotide polymorphisms (SNPs) as instrumental variables to accurately reflect the phenotypes of exposure and outcome components, thereby inferring the causal relationship between them [18]. Since the medium genes are assigned at random during fertilization, the likelihood of inheritance is equitably distributed among individuals, analogizing the procedure to randomized controlled trials (RCTs). This analogy helps to mitigate selection and information bias in observational research. Furthermore, MR Has the advantages of effectively reducing confounder bias, large sample size, more reliable results, and less possibility of reverse causality [19–21]. Hence, MR emerges as an optimal research approach for unraveling disease risk factors and causation. Wang used MR to study the bidirectional causal relationship between IBD and periodontitis, and Bae explored the unidirectional causal relationship between periodontitis and SLE and arthritis [22, 23]. However, the current studies are not comprehensive enough, either only studying one-way potential causation, or only studying the relationship between a single disease and periodontitis, selecting a single database, and failing to conduct comprehensive analysis of the combined results of multiple databases.

The bidirectional pathogenic relationship between IMIDs and periodontal disease has not been confirmed. Therefore, we hypothesize that there is a causal relationship between IMIDs and periodontal disease. This study utilized the statistical data of genome-wide association studies (GWAS) to select ten common IMIDs and verified the causal association between IMIDs and periodontal disease using the bidirectional two-sample MR method. In addition, this study employed multiple public databases for repeated verification, maximizing the reliability of the results. Our study aimed to provide a reliable basis for the clinical formulation of feasible prevention and treatment strategies for periodontal disease and IMIDs, and at the same time, promote interdisciplinary collaboration between clinicians and stomatologists, facilitating diligent screening, effective prevention, and timely intervention across multiple specialties.

## Materials and methods

### Study design

**Fig. 1:**
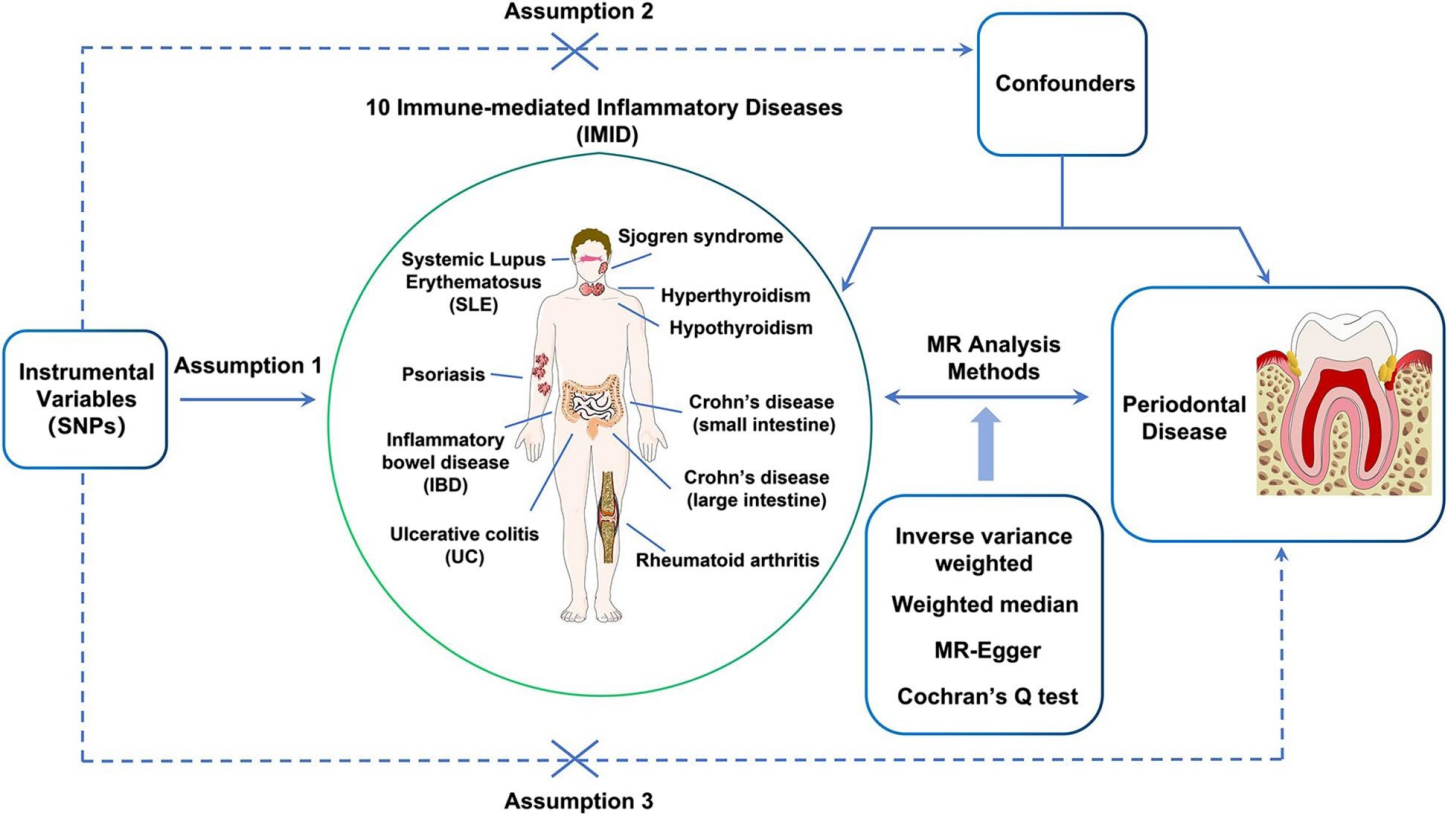
The flowchart illustrating of Mendelian randomization study design. Assumption 1, genetic variants are strong correlated with exposure, IMIDs and periodontal disease acted as exposures and outcomes of each other, respectively; Assumption 2, genetic variants are independent of confounding factors associated with exposure and outcome; Assumption 3, genetic variants influence outcome only through the exposure. IMID, Immune-mediated inflammatory disorders; MR, Mendelian randomization; SNPs, Single nucleotide polymorphisms; SLE, Systemic lupus erythematosus; IBD, Inflammatory bowel disease; UC, Ulcerative colitis

We comprehensively evaluated the causal association between ten kinds of IMID and the risk of periodontal disease, utilizing a bidirectional two-sample MR design. The ten IMIDs included hyperthyroidism, hypothyroidism, SLE, Crohn’s disease (small intestine), Crohn’s disease (large intestine), IBD, UC, psoriasis, rheumatoid arthritis, and Sjogren syndrome. The flowchart illustrating our study design is depicted in Fig. 1. In our analysis, IMIDs and periodontal disease acted as exposures and outcomes of each other, respectively. The instrument variables associated with the exposures were obtained and harmonized with relevant information from the outcomes. Subsequently, three distinct MR analysis methods and sensitivity analyses were applied. To establish the credibility of the MR analysis, three key presumptions must be met: (1) the genetic instruments exhibit robust correlation with the exposures under investigation; (2) the genetic instruments are not correlated with confounding factors; and (3) the genetic instruments solely affect the outcome through the exposures of interest [24]. To address the primary and tertiary points, we implemented a rigorous methodology in our study, including selecting strong variables, identifying pertinent confounders through PhenoScanner, and utilizing MR-Egger intercept analysis to assess potential pleiotropy [25]. As for the second point, since SNPs are randomly assigned in the process of meiosis, this study has fulfilled the requirements. Genetic data on periodontal disease originated from three distinct GWAS consortia, while IMID genetic information was derived from two independent GWAS consortia. These datasets underwent primary analysis, sensitivity analyses, replication, and finally a meta-analysis.

All statistical analyses were conducted utilizing the "TwoSampleMR" package (Version 0.5.4) in the R program (Version 4.0.0).

### GWAS data for ten kinds of IMID

We utilized publicly available GWAS aggregate statistics for various IMIDs acquired from the FinnGen Consortium on May 31, 2023, and the UK Biobank (UKB) on June 9, 2023. R9 GWAS data was published by the FinnGen Consortium (https://r9.finngen.fi/), and GWAS round 2 data was published by the UKB (http://www.nealelab.is/uk-biobank). The FinnGen Consortium provided data on cases and controls, ranging from 1023 to 36321 and 240862 to 371530. Similarly, the UKB exhibited a range of cases and controls varying from 150 to 17574 and 343567 to 360991. For specific figures, please refer to additional file 1, Table S1. All populations in this study exclusively comprise individuals of European ancestry to mitigate potential bias from demographic heterogeneity.

### GWAS data for periodontal disease

Publicly available GWAS aggregate statistics for periodontal disease were employed, obtained from the FinnGen Consortium on June 6, 2023, UKB on June 9, 2023, and the Gene Lifestyle Interactions in Dental Endpoints (GLIDE) consortium on July 14, 2023. The numbers of cases and controls were 87497 and 259234 in the FinnGen Consortium, respectively. As for the UKB dataset, it consisted of 458 cases and 360736 controls for periodontal disease. In addition, the GLIDE consortium contributed clinically diagnosed periodontitis GWAS data, including 17,353 periodontitis cases and 28,210 controls [26]. Only individuals of European ancestry were included in the study to address demographic heterogeneity.

### Instruments selection

When considering the ten IMIDs as exposure factors, we utilized SNPs that exhibited a significant association with exposure at a threshold of *P* < 5 × 10^-8^. To reduce collinearity, we calculated pairwise linkage disequilibrium (LD), SNPs were excluded with *r*^2^ ≥ 0.001 and LD distance ≤ 10,000 kb. To minimize bias due to weak instruments, SNPs were evaluated using the F-statistic for statistical robustness. Those with an F-value below 10 were removed [27, 28]. Next, the SNP associated with the exposure was extracted from the outcome data, while the one associated with the outcome was excluded. Finally, IMIDs with more than two SNPs were retained for MR analysis.

When utilizing periodontal disease as an exposure factor, we selected the SNPs associated with corresponding exposure and set their effective threshold at *P* < 5 × 10^-6^. Due to the limited availability of SNPs reaching genome-wide significance (*P* < 5 × 10^-8^), this more lenient threshold has been widely applied in various studies [21, 29]. By calculating pairwise linkage disequilibrium, we excluded SNPs with *r*^2^ ≥ 0.1 and LD distance ≤ 500 kb. The subsequent steps are the same when IMIDs is used as the factor of exposure.

The F-statistic for each SNP was computed using the formula: F = *R*^2^ / (1 − *R*^2^) * (N-2). In the formula, *R*^2^ represents variance of exposure explained by instrument variable, while N denotes the sample size. Moreover, we determined the variance in exposure explained by the instrument variable using the following formula: *R*^2^ = β^2^ / (β^2^ + se^2^ *N). Within this equation, β denotes the effect size of the specific genetic variant, se represents the standard error of β, and N indicates the sample size.

### Primary analysis

In the primary analyses, we assessed the association between IMID (sourced from the FinnGen Consortium) and periodontal disease (obtained from the GLIDE consortium). In our investigation, MR analyses utilized the commonly employed inverse variance weighted (IVW) method to generate a pooled estimate by combining Wald ratios from each SNP. IVW was used as the major approach in this investigation to investigate early links between IMID and periodontal disease, with a significance level set at *P* < 0.05.

### Mendelian randomization and sensitivity analyses

The main analysis employed random effect IVW since it has robustness and the ability to provide reliable estimates in the presence of heterogeneity. However, unbiased estimates of causal effects can only be obtained if all SNPs are valid instrumental variables and not influenced by unbalanced horizontal pleiotropy [30]. To explore sensitivity and ensure the reliability of the findings, we conducted complementary sensitivity analyses using the MR-Egger and weighted median (WM) methods. The MR-Egger regression approach allowed us to identify potential imbalanced pleiotropy and significant heterogeneity [31]. The WM estimates were utilized to generate robust impact estimates, ensuring that at least half of the weighted variance, weighted by horizontal pleiotropy, was accurate [32]. Specifically, an IVW significant estimate was considered to be significant if it aligned with the trends in the estimates derived from WM and MR-Egger. The Cochran's Q tests, funnel plots, leave-one-out analyses, and MR-Egger intercept tests were also carried out. More specifically, Cochran’s Q tests were used to identify the presence of heterogeneity, with a significant level of *P* < 0.05 recognized indicative of heterogeneity [33]. Pleiotropy was assessed by using the MR-Egger regression’s intercept term [31, 33].

All estimates were presented as odds ratios (OR) for each additional standard deviation (SD) of the relevant exposure.

### Replication and meta‑analysis

There may be some inconsistency in the results of previous studies, which are mainly limited by population selection. Therefore, in order to verify whether these results are consistent across different populations, we did repeat validation across multiple databases. We carried out a series of tests to validate the robustness of the candidate exposure variables: 1) When ten IMIDs were used as exposure factors, we repeated the IVW analysis to reveal the causal effect between IMID from the UKB and periodontal disease from the FinnGen Consortium and GLIDE consortium. To elucidate the origin of the database for exposure and outcome, we named the database “Exposure to Outcome”. Specifically, we employed UKB to FinnGen, and UKB to GLIDE, to ensure no overlap between databases. 2) When periodontal disease were used as exposure factors, we also repeated the IVW analysis using periodontal disease data from the UKB and FinnGen Consortium as well as IMID data from the UKB and FinnGen Consortium. FinnGen to UKB and UKB to FinnGen were utilized to ensure that the databases didn’t overlap. Finally, a meta-analysis methodology was employed to aggregate findings from diverse databases and validate the ultimate causal relationship.

## Results

In this study, we thoroughly screened multiple instrumental variables based on the instrumental variable selection protocol. This included the selection of 3 to 131 SNPs as variable tools for IMIDs (see additional file 2, Table S2), as well as 8, 23, and 44 SNPs as instrument variables for periodontal disease from different databases (additional file 3, Table S3). The F-statistic values of all SNPs exceeded 10, indicating that no weak instrumental variables were used. The values of MR analysis for different databases with different methods were shown in additional file 4 and 5, Table S4 and Table S5.

### Primary analysis

#### Causal effects of IMID (FinnGen) to periodontal disease (GLIDE)

A causal relationship between SLE and periodontal disease was observed in the primary IVW outcome, suggesting an increased risk of periodontal disease casually related to SLE [IVW: OR = 1.079 (95% CI: 1.032-1.128) and *P* < 0.001]. The aforementioned finding is further confirmed by WM analyses and MR-Egger analysis [WM: OR = 1.067 (95% CI: 1.010-1.127) and *P* = 0.021; MR-Egger: OR = 1.000 (95% CI: 0.896-1.116) and *P* = 0.994]. The study also demonstrated a causal association between Sjogren syndrome and enhanced risk of periodontal disease [IVW: OR = 1.082 (95% CI: 1.012-1.157) and *P* = 0.022]. WM analysis provided consistent and significant outcomes [WM: OR = 1.072 (95% CI: 1.003-1.145) and *P* = 0.041], whereas MR-Egger analysis showed no significance [MR-Egger: OR = 0.985 (95% CI: 0.879-1.104) and *P* = 0.808]. Since the directions of the three methods were inconsistent and the robustness of causality was limited, caution should be paid in interpreting the potential causal effects of Sjogren syndrome.

**Fig. 2:**
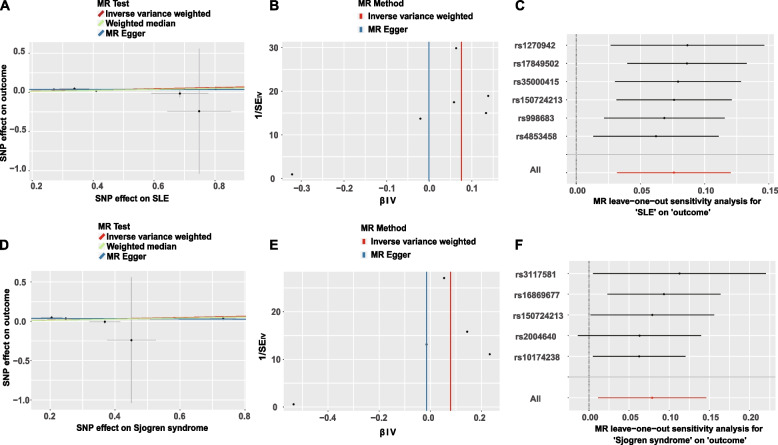
Three kinds of plots for the causal relationship between IMID and periodontal disease. **A** scatter plot of SLE using different MR methods; **B** funnel plot of SLE; **C** leave-one-out plot of SLE; **D** scatter plot of Sjogren syndrome using different MR methods.; **E** funnel plot of Sjogren syndrome; **F** leave-one-out plot of Sjogren syndrome. IMID, Immune-mediated inflammatory disorders; MR, Mendelian randomization; SNP, Single nucleotide polymorphism; SLE, Systemic lupus erythematosus

The values of MR analysis for IVW were presented in Table 1. The aforementioned causal estimates were verified using the funnel, scatter, and leave-one-out plot analyses (refer to Fig. 2).

**Table 1:** MR analysis of IVW for IMIDs to periodontal disease

**Database (Exposure to Outcome)**	**Exposure**	**OR**	**CI up**	**CI low**	***P*-value**
**FinnGen to GLIDE**	***Hyperthyroidism***	0.977	0.935	1.020	0.280
	***Hypothyroidism***	0.990	0.946	1.036	0.650
	***SLE***	1.079	1.032	1.128	< 0.001
	***Crohn’s disease (small intestine)***	1.020	0.939	1.109	0.633
	***Crohn’s disease (large intestine)***	0.968	0.889	1.054	0.457
	***IBD***	0.984	0.942	1.028	0.473
	***UC***	0.981	0.943	1.022	0.358
	***Psoriasis***	1.015	0.953	1.080	0.644
	***Rheumatoid arthritis***	1.028	0.953	1.109	0.472
	***Sjogren syndrome***	1.082	1.012	1.157	0.022
**UKB to FinnGen**	***Hyperthyroidism***	6.40	6.34*10^-2^	6.45*10^2^	0.430
	***Hypothyroidism***	1.52	1.13	2.04	0.005
	***SLE***	7.57	5.70*10^-3^	1.00*10^4^	0.581
	***Crohn’s disease (small intestine)***	9.58*10^-2^	6.80*10^-4^	1.35*10	0.353
	***Crohn’s disease (large intestine)***	2.90*10^-1^	7.04*10^-5^	1.19*10^3^	0.771
	***IBD***	3.38*10^-1^	4.96*10^-2^	2.31	0.268
	***UC***	1.70	1.39*10^-2^	2.09*10^2^	0.828
	***Psoriasis***	1.26	2.11*10^-1^	7.55	0.799
	***Rheumatoid arthritis***	7.06*10^-1^	2.19*10^-3^	2.28*10^2^	0.906
**UKB to GLIDE**	***Hyperthyroidism***	3.21*10^-4^	5.80*10^-8^	1.78	0.067
	***Hypothyroidism***	8.91*10^-1^	3.59*10^-1^	2.21	0.803
	***SLE***	1.69	1.86*10^-8^	1.53*10^8^	0.955
	***Crohn’s disease (small intestine)***	1.58*10	1.07*10^-15^	2.33*10^17^	0.885
	***Crohn’s disease (large intestine)***	3.32*10^-2^	9.56*10^-13^	1.16*10^9^	0.783
	***IBD***	4.75*10^-3^	1.72*10^-9^	1.31*10^4^	0.480
	***UC***	6.48*10^-5^	3.93*10^-11^	1.07*10^2^	0.187
	***Psoriasis***	1.57	2.01*10^-2^	1.23*10^2^	0.839
	***Rheumatoid arthritis***	1.68*10^-1^	1.61*10^-3^	1.75*10	0.452

*IVW* Inverse variance weighted, *MR* Mendelian randomization, *IMID* Immune-mediated inflammatory disorders, *OR* Odds ratios, *CI* Confidence interval, *UKB* UK Biobank, *GLIDE* Gene Lifestyle Interactions in Dental Endpoints, *SLE* Systemic lupus erythematosus, *IBD* Inflammatory bowel disease, *UC* Ulcerative colitis

#### Causal effects of periodontal disease (GLIDE) to IMID (FinnGen)

The primary IVW outcome [IVW: OR = 0.8079 (95% CI: 0.6764-0.9650) and *P* = 0.019] indicated a potential causal association between periodontal disease and SLE. This finding was further reinforced by WM analyses [WM: OR = 0.8072 (95% CI: 0.6452-1.0097) and *P* = 0.061] and MR-Egger analysis [MR-Egger: OR = 0.7636 (95% CI: 0.6222-0.9370) and *P* = 0.061]. The estimates implied that periodontal disease might reduce the risk of SLE.

The values of MR analysis for IVW were presented in Table 2. To validate the previously established causal estimations, the funnel plot, scatter plot, and leave-one-out plot were employed in the study (refer to additional file 6, Figure S1).

**Table 2:** MR analysis of IVW for periodontal disease to IMIDs

**Database (Exposure to Outcome)**	**Outcome**	**OR**	**CI up**	**CI low**	***P*-value**
**FinnGen to UKB**	***Hyperthyroidism***	0.9991	0.9966	1.0016	0.467
	***Hypothyroidism***	1.0015	0.9940	1.0091	0.694
	***SLE***	0.9997	0.9987	1.0006	0.508
	***Crohn’s disease (small intestine)***	1.0003	0.9995	1.0011	0.477
	***Crohn’s disease (large intestine)***	1.0004	0.9995	1.0013	0.354
	***IBD***	1.0004	0.9997	1.0010	0.266
	***UC***	1.0010	0.9984	1.0036	0.449
	***Psoriasis***	1.0024	0.9993	1.0054	0.133
	***Rheumatoid arthritis***	1.0016	0.9979	1.0053	0.392
	***Sjogren syndrome***	0.9992	0.9983	1.0001	0.067
**GLIDE to FinnGen**	***Hyperthyroidism***	0.9563	0.8219	1.1126	0.563
	***Hypothyroidism***	1.0173	0.9771	1.0592	0.404
	***SLE***	0.8079	0.6764	0.9650	0.019
	***Crohn’s disease (small intestine)***	0.9422	0.8152	1.0890	0.420
	***Crohn’s disease (large intestine)***	1.0607	0.9007	1.2492	0.480
	***IBD***	1.0288	0.9531	1.1106	0.466
	***UC***	1.0687	0.9744	1.1720	0.158
	***Psoriasis***	1.0065	0.9404	1.0771	0.853
	***Rheumatoid arthritis***	0.9555	0.8816	1.0356	0.268
	***Sjogren syndrome***	0.9774	0.8428	1.1336	0.763
**UKB to FinnGen**	***Hyperthyroidism***	5.59*10^-9^	1.43*10^-15^	2.18*10^-2^	0.014
	***Hypothyroidism***	3.98	1.16*10^-1^	1.37*10^2^	0.444
	***SLE***	3.47*10^-5^	4.88*10^-14^	2.47*10^4^	0.323
	***Crohn’s disease (small intestine)***	5.12*10^3^	1.48*10^-3^	1.78*10^10^	0.266
	***Crohn’s disease (large intestine)***	7.93*10^6^	2.10*10^-1^	2.99*10^14^	0.074
	***IBD***	2.43*10^-2^	2.26*10^-6^	2.61*10^2^	0.432
	***UC***	1.77*10^-3^	1.92*10^-7^	1.62*10	0.173
	***Psoriasis***	2.71*10^-1^	3.07*10^-4^	2.40*10^2^	0.706
	***Rheumatoid arthritis***	5.72*10^-1^	1.02*10^-3^	3.22*10^2^	0.863
	***Sjogren syndrome***	2.10*10^-2^	5.05*10^-8^	8.76*10^3^	0.559

*IVW* Inverse variance weighted, *MR* Mendelian randomization, *IMID* Immune-mediated inflammatory disorders, *OR* Odds ratios, *CI* Confidence interval, *UKB* UK Biobank, *GLIDE* Gene Lifestyle Interactions in Dental Endpoints, *SLE* Systemic lupus erythematosus, *IBD* Inflammatory bowel disease, *UC* Ulcerative colitis

#### Sensitivity analyses and detection of pleiotropy

The Cochran Q test results revealed no significant heterogeneity (*p* > 0.05) (refer to Table 3). In order to avoid an excessive bias effect, we also conducted a pleiotropic analysis as presented in Table 3. There was no polymorphism in IMID causally associated with periodontal disease (*p* > 0.05) (Table 3 is at the end of the article).

**Table 3:** Sensitivity analyses and detection of pleiotropy

**Database (Exposure to Outcome)**	**Exposure**	**Intercept**	***p*-value**	**Q**	**Q_ *p*-value**
**FinnGen to GLIDE**	***Hyperthyroidism***	-0.029	0.127	9.331	0.407
	***Hypothyroidism***	-0.001	0.881	131.862	0.121
	***SLE***	0.037	0.210	4.269	0.511
	***Crohn’s disease (small intestine)***	0.018	0.621	14.266	0.047
	***Crohn’s disease (large intestine)***	0.005	0.932	0.512	0.774
	***IBD***	0.015	0.119	25.568	0.819
	***UC***	0.011	0.340	28.532	0.594
	***Psoriasis***	0.009	0.520	17.122	0.803
	***Rheumatoid arthritis***	0.003	0.812	30.585	0.081
	***Sjogren syndrome***	0.039	0.162	6.014	0.198
**UKB to FinnGen**	***Hyperthyroidism***	-0.008	0.926	4.282	0.118
	***Hypothyroidism***	0.001	0.639	75.287	0.502
	***SLE***	-0.011	0.438	2.082	0.556
	***Crohn’s disease (small intestine)***	-0.015	0.548	6.432	0.377
	***Crohn’s disease (large intestine)***	-0.008	0.787	1.954	0.582
	***IBD***	-0.001	0.902	2.213	0.819
	***UC***	0.009	0.384	5.734	0.333
	***Psoriasis***	0.000	0.959	22.580	0.007
	***Rheumatoid arthritis***	-0.004	0.895	9.717	0.021
**UKB to GLIDE**	***Hyperthyroidism***	-0.027	0.785	3.383	0.496
	***Hypothyroidism***	0.001	0.850	98.676	0.101
	***SLE***	0.043	0.344	5.180	0.159
	***Crohn’s disease (small intestine)***	-0.018	0.900	6.275	0.280
	***Crohn’s disease (large intestine)***	0.011	0.933	4.556	0.207
	***IBD***	-0.002	0.958	1.263	0.868
	***UC***	0.041	0.389	9.090	0.246
	***Psoriasis***	-0.009	0.449	20.242	0.042
	***Rheumatoid arthritis***	-0.021	0.349	4.608	0.330
**Database (Exposure to Outcome)**	**Outcome**	**intercept**	***p*-value**	**Q**	**Q_ *p*-value**
**FinnGen to UKB**	***Hyperthyroidism***	-3.24*10^-5^	0.770	8.591	0.929
	***Hypothyroidism***	2.67*10^-4^	0.438	24.479	0.080
	***SLE***	-2.29*10^-5^	0.599	10.432	0.843
	***Crohn’s disease (small intestine)***	-5.19*10^-5^	0.163	19.703	0.234
	***Crohn’s disease (large intestine)***	-1.66*10^-5^	0.670	5.340	0.994
	***IBD***	3.12*10^-5^	0.284	19.483	0.244
	***UC***	1.72*10^-5^	0.887	25.406	0.063
	***Psoriasis***	1.43*10^-5^	0.918	15.450	0.492
	***Rheumatoid arthritis***	-2.50*10^-4^	0.124	23.828	0.093
	***Sjogren syndrome***	2.18*10^-5^	0.580	8.743	0.924
**GLIDE to FinnGen**	***Hyperthyroidism***	-4.72*10^-4^	0.954	6.351	0.274
	***Hypothyroidism***	-4.72*10^-4^	0.954	6.351	0.274
	***SLE***	3.70*10^-2^	0.337	4.476	0.483
	***Crohn’s disease (small intestine)***	-1.96*10^-3^	0.941	1.245	0.940
	***Crohn’s disease (large intestine)***	-2.93*10^-2^	0.351	1.774	0.879
	***IBD***	-9.42*10^-3^	0.529	5.143	0.399
	***UC***	-2.14*10^-2^	0.244	4.312	0.505
	***Psoriasis***	6.06*10^-3^	0.629	1.949	0.856
	***Rheumatoid arthritis***	6.12*10^-3^	0.702	8.723	0.121
	***Sjogren syndrome***	7.28*10^-3^	0.807	6.425	0.267
**UKB to FinnGen**	***Hyperthyroidism***	3.53*10^-3^	0.534	21.445	0.921
	***Hypothyroidism***	3.53*10^-3^	0.534	21.445	0.921
	***SLE***	-1.02*10^-2^	0.751	21.679	0.916
	***Crohn’s disease (small intestine)***	-6.50*10^-3^	0.789	34.603	0.345
	***Crohn’s disease (large intestine)***	-1.90*10^-3^	0.947	37.155	0.243
	***IBD***	2.07*10^-3^	0.891	49.716	0.024
	***UC***	-1.82*10^-3^	0.901	31.234	0.505
	***Psoriasis***	8.10*10^-3^	0.457	27.513	0.693
	***Rheumatoid arthritis***	1.37*10^-2^	0.178	35.841	0.293
	***Sjogren syndrome***	-2.23*10^-2^	0.286	21.678	0.916

*UKB* UK Biobank, *GLIDE* Gene Lifestyle Interactions in Dental Endpoints, *SLE* Systemic lupus erythematosus, *IBD* Inflammatory bowel disease, *UC* Ulcerative colitis, *Q* heterogeneity statistic Q

#### Replication and meta‑analysis

When ten IMIDs were utilized as exposure factors, we repeated the analysis using the UKB. The MR analysis conducted on the IMID (UKB) to periodontal disease (GLIDE) suggested negative results. Hence, none of the ten IMIDs had a causal effect on periodontal disease. In contrast to the primary analysis, no causal relationship was observed between SLE and periodontal disease [IVW: OR = 1.69 (95% CI: 1.86*10^–8^-1.53*10^8^) and *P* = 0.955]. Conversely, when analyzing the association between IMIDs (UKB) and periodontal disease (FinnGen), a causal effect of hypothyroidism on the increased risk of periodontal disease was identified [IVW: OR = 1.52 (95% CI: 1.13-2.04) and *P* = 0.005]. This finding was further substantiated by WM analyses [OR = 1.37 (95% CI: 8.69*10^-1^-2.18) and *P* = 0.174]. The MR-Egger analysis assessing causality also indicated a consistent correlation, although it did not reach statistical significance [OR = 1.34 (95% CI: 7.24*10^-1^-2.47) and *P* = 0.357]. The findings of the funnel plot, scatter plot, and leave-one-out plot can be found in additional file 7 and 8, Figure S2 and Figure S3.

When periodontal disease was utilized as an exposure factor, we repeated the analysis using the FinnGen and UKB. The MR analysis conducted on the periodontal disease (UKB) to IMID (FinnGen) revealed that periodontal disease might reduce the risk of hyperthyroidism [IVW: OR = 5.59*10^-9^ (95% CI: 1.43*10^-15^-2.18*10^-2^) and *P* = 0.014]. WM analysis and MR-Egger analysis presented consistent outcomes [WM: OR = 1.92*10^-2^ (95% CI: 8.96*10^-12^-4.12*10^7^) and *P* = 0.718] and [MR-Egger: OR = 7.06*10^-8^ (95% CI: 1.04*10^-17^-4.81*10^2^) and *P* = 0.164]. Additional file 6, Figure S1 displayed the funnel plot, scatter plot, and leave-one-out plot. The results of the MR analysis in the periodontal disease (FinnGen) to IMID (UKB) were all negative, and the periodontal disease had no causal relationship with any of the ten IMIDs.

**Fig. 3:**
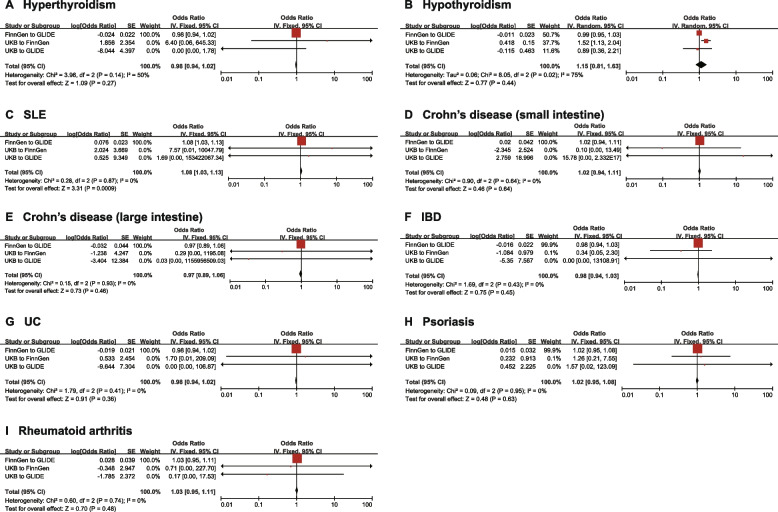
Meta-analysis of the causal effects of IMIDs to periodontal disease on different databases. To elucidate the origin of the database for exposure and outcome, we named the database “Exposure to Outcome”. As shown in the figure, we employed FinnGen to GLIDE, UKB to FinnGen, and UKB to GLIDE, to ensure no overlap between databases. IMID, immune-mediated inflammatory disorders; CI, confidence interval; UKB, UK Biobank; GLIDE, Gene Lifestyle Interactions in Dental Endpoints; SLE, Systemic lupus erythematosus; IBD, Inflammatory bowel disease; UC, Ulcerative colitis

**Fig. 4:**
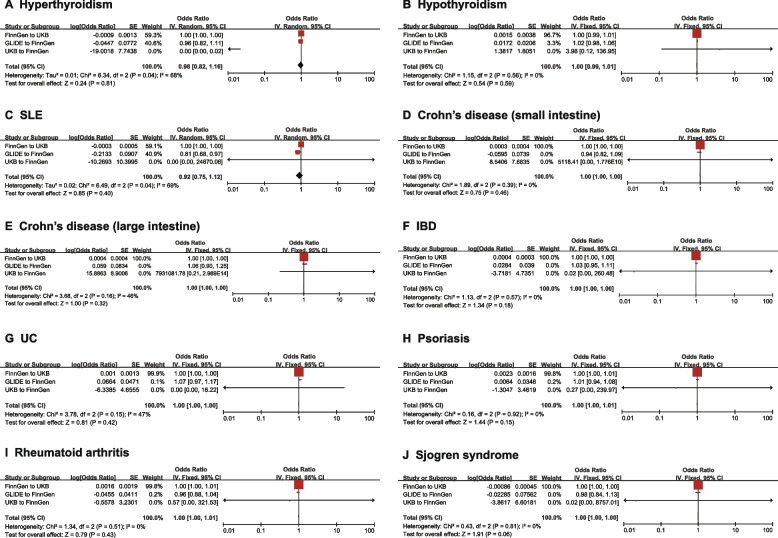
Meta-analysis of the causal effects of periodontal disease to IMIDs on different databases. To elucidate the origin of the database for exposure and outcome, we named the database “Exposure to Outcome”. As shown in the figure, we employed FinnGen to UKB, GLIDE to FinnGen, and UKB to FinnGen, to ensure no overlap between databases. IMID, immune-mediated inflammatory disorders; CI, confidence interval; UKB, UK Biobank; GLIDE, Gene Lifestyle Interactions in Dental Endpoints; SLE, Systemic lupus erythematosus; IBD, Inflammatory bowel disease; UC, Ulcerative colitis

A comprehensive meta-analysis of the database was conducted to consolidate and further validate the results, indicating a causal correlation between SLE and an increased risk of periodontal disease: [OR = 1.08 (95% CI: 1.03-1.13), *I*^2^ = 0%, *P* = 0.0009]. Furthermore, the other nine types of IMID exhibited no bidirectional causal connection with periodontal disease according to the results of the meta-analysis (refer to Figs. 3 and 4).

## Discussion

We implemented a multi-faceted approach to investigate the causal association between IMIDs and periodontal disease. This included conducting estimations using diverse analytical methods such as IVW, MR-Egger, and WM and validating the findings through analysis across multiple databases and meta-analysis summaries. Through two-way and two-sample MR research analysis, our study ultimately concluded that SLE could significantly elevate the risk of periodontal disease.

A recent systematic review and meta-analysis highlighted a bidirectional causal association between periodontal disease and SLE. This comprehensive analysis primarily focused on assessing the prevalence of periodontal disease among SLE patients. Moreover, additional outcomes encompassing probing depth, loss of attachment, and disease activity index, among others, were meticulously examined, ultimately unveiling the heightened susceptibility of patients with SLE to the onset of periodontal disease [34]. Rutter-Locher et al. conducted a systematic review comprising eight case-control studies involving 1383 participants, of which 487 were cases. The study findings revealed that patients with SLE exhibited a significant elevation in the risk of periodontitis compared to control groups [35]. Furthermore, within a population-based study conducted in Norway, 1,990 patients from the SLE cohort were recruited. In this investigation, periodontitis was employed as the outcome measure for logistic regression analysis, allowing the estimation of odds ratios between patients with periodontitis and a control group. The findings revealed a notably heightened risk of periodontitis among SLE patients, nearly doubling that of the control group. This discrepancy was particularly prominent in younger individuals [36]. These findings corroborate the aforementioned results of our study, thus offering added validation for the causal association between SLE and periodontal disease.

Several studies have elucidated potential mechanistic pathways that support the association between SLE and periodontal disease [37]. Firstly, to investigate the potential influence of systemic immune disorder on subgingival microbiota, a study assessed the impact of SLE on subgingival microbiota and observed a marked increase in the bacterial load among SLE patients. This rise in pathogen abundance was accompanied by a substantial alteration in the overall structure of bacterial composition [38]. Other studies have shown a relationship between SLE activity and severity and changes in periodontal disease-associated microbiota [39]. Moreover, an imbalance in pro-inflammatory and anti-inflammatory cytokines induced by SLE seemed to cause tissue damage [38]. Marques, C. P. et al. conducted a case-control study, enrolling 60 SLE patients and 65 healthy individuals. A comprehensive periodontal clinical examination was conducted on all participants, revealing elevated salivary cytokines in both periodontal disease patients and SLE patients within the oral cavity [40]. Salivary IL-1β and IL-4 levels positively correlated with periodontitis severity, suggesting their potential as markers for assessing periodontal damage in SLE patients. Finally, evidence also indicated activation of autoreactive B cells and dysregulation of immune cell populations (macrophages, neutrophils, CD4 + T cells, and dendritic cells) may be the underlying mechanisms [41]. In addition, some scholars believed that the expression of toll (TLR-2 and TLR-4) -like receptors was elevated in both SLE and periodontal disease. Periodontitis might induce the overactivation of the immune response in SLE by maintaining high expression of TLR, which would then lead to the accelerated occurrence and progression of the autoimmune response [42]. Furthermore, periodontal therapy has been shown to decrease the expression of these receptors, thereby alleviating the symptoms of SLE [42]. In clinical practice, it is imperative to enhance patients' comprehension of the significance of periodontal therapy for individuals with SLE while promoting their active involvement in managing their periodontal health. By doing so, it is possible to effectively reduce the expression of toll-like receptors, subsequently mitigating certain symptoms associated with SLE. Significantly, our study also highlighted the potential impact of periodontal disease in reducing the risk of SLE. However, following rigorous validation using diverse databases and meta-summary analysis, this study concluded that periodontal disease does not promote or inhibit SLE.

This study utilized MR analysis of a single database, revealing the contribution of Sjogren's syndrome and hypothyroidism to promoting periodontal disease. Nonetheless, thorough validation and meta-summary analyses across diverse databases demonstrated the absence of a causal link between these two immune diseases and periodontal disease. Sjogren's syndrome predominantly affects the body's exocrine glands, including the lacrimal and salivary glands [43]. As Sjogren’s syndrome advances, there is a gradual decrease in saliva production, which may have implications for the oral periodontal tissues. While diminished saliva flow can adversely affect oral health, a direct causal relationship between Sjogren’s syndrome and periodontal disease remains unestablished [43, 44]. In other investigations, the presence of a definitive causal association between Sjogren's syndrome and periodontal disease has not been firmly established. While it appears that individuals with Sjogren's syndrome have a higher likelihood of being diagnosed with periodontal disease, caution must be exercised in drawing conclusive findings due to the substantial heterogeneity observed in the studies [45].

Limited high-quality studies have shown a positive correlation between hypothyroidism and periodontal disease, with minimal confounding variables considered. However, further well-controlled prospective clinical and immunological investigations are needed to confirm this relationship and control potential confounders [46]. Reduced thyroid hormone levels impair bone metabolism, maturation, and turnover, consequently negatively influencing bone homeostasis [47]. Feitosa et al. conducted an experimental study where implants were surgically placed in the tibia of rats with hypothyroidism. The findings demonstrated that thyroid hormone might impact the healing process of cortical bone surrounding titanium implants in rats. Specifically, the study observed a significant reduction in the new bone area and bone density surrounding the implants [48, 49]. Our discovery augments the existing genetic evidence by elucidating that hypothyroidism elevates the susceptibility to periodontal disease.

Furthermore, our study also observed a potential inverse relationship, suggesting that periodontal disease may impede the risk of hyperthyroidism. Nonetheless, there is a lack of literature documenting the correlation between periodontal disease and hyperthyroidism. Additional investigations are warranted to substantiate the existence of any association or causal relationship between periodontal disease and hyperthyroidism. The aforementioned conclusions should be corroborated through further clinical, experimental, and mechanistic studies. Despite repeated validation and meta-analysis, our results' lack of significant findings may be attributed to the heterogeneity arising from variations in countries, sample sizes, and database quality across different studies.

Moreover, no causal link has been established between other IMIDs and periodontal disease. Published Mendelian randomization studies yield varying results, with some supporting our findings and others not. Some investigations suggest a weak bidirectional causal association between IBD, UC, Crohn's disease, and periodontitis; however, the clinical relevance of these associations may be limited [22]. Certain studies propose a tenuous causal link between periodontitis and rheumatoid arthritis [23]. These findings merely suggest a tenuous causal link between the two variables, necessitating additional research for substantiation. The disparity between our study's outcomes and the aforementioned studies may arise from database and groups selection variations. Notably, our study has a larger sample size and adopts repeated validation and meta-analysis, thereby rendering our outcomes more convincing. Although many observational studies and reviews have shown an association between IBD [50], UC [51], Crohn's disease [52], psoriasis [53, 54], rheumatoid disease [55], and periodontal disease, our results imply that the causal relationship between the diseases is not firmly supported. Consequently, further exploration of disease interactions and pathogenesis is needed in the future.

Our research exhibits notable strengths. Firstly, the implementation of the MR method minimizes the impact of reverse causality and confounding variables. Moreover, our MR studies encompass a wide range of individuals while maintaining cost-effectiveness, while the large sample sizes may be more practical and persuasive than traditional observational studies. Moreover, we performed iterative analyses and meta-analyses utilizing various independent databases to validate the robustness of our MR estimates, thereby significantly bolstering confidence in our findings. However, it is also important to acknowledge several limitations in our study. Due to the restricted number of SNPs achieving genome-wide significance, we employed a commonly employed methodology to expand the P-threshold for periodontal disease as an exposure variable. In future research, we should try our best to select a database with an appropriate threshold value for analysis to make the research more rigorous. Secondly, the majority of participants in this study were of European descent, which might limit the generalizability of our findings to other populations. While this choice helps mitigate population heterogeneity, validating the MR results in additional populations is crucial to ensuring generalizability. This can be achieved by including GWAS data from diverse populations in future studies when such data becomes publicly accessible. Finally, despite the outstanding capability of the MR method in causal inference. However, because MR Studies may be limited by factors such as developmental compensation, biological complexity and phenotypic heterogeneity, which affect the application of MR Studies in causal inference [56, 57], it is important to underscore the necessity of corroborating the findings of this MR study with a large number of rigorous randomized controlled trials. This validation step is crucial to establishing a genuine causal relationship.

Our research will help to provide a reliable basis for formulating feasible prevention and treatment strategies for periodontal disease and IMIDs, promote interdisciplinary cooperation between clinicians and stomatologists, increase doctors' awareness of diagnosis and treatment, and promote careful screening, effective prevention and timely intervention among multiple specialties. When receiving patients, clinicians in the department of immunology and rheumatology can ask patients to pay more attention to periodontal health, encourage patients to undergo related genetic testing, investigate related pathogenic genes, and go to the department of stomatology for examination and early intervention, which is conducive to the maintenance of periodontal health and prevents or delays the occurrence and development of periodontal diseases. The dentist should pay more attention to the systemic condition of some patients when receiving patients with periodontitis, which is conducive to the early detection and treatment of the disease.

## Conclusions

In summary, this study demonstrated the causal relationship between IMID and periodontal disease in through MR analysis and revealed the bidirectional influence between ten kinds of IMIDs and the risk of periodontal disease. SLE, Sjogren syndrome, and hypothyroidism might increase the risk of periodontal disease. In addition, periodontal disease might reduce the risk of SLE and hyperthyroidism. However, no bilateral causal relationship was found between other IMIDs and periodontal disease. Only SLE was confirmed to be associated with an increased risk of periodontal disease after meta-analysis. Although our final results didn’t support a causal relationship between periodontal disease and the risk of IMID, further research is needed to clarify the effects of periodontal disease on IMID, and larger sample sizes or more studies are needed to generalize to other populations. Our study can potentially influence clinical decisions on periodontal management in patients with IMID, providing a new direction for clinical intervention in periodontal disease, and promotes serious screening, effective prevention and timely intervention among multiple professions, and is conducive to early detection and treatment of diseases.

### Supplementary Information

Supplementary Material 1.

Supplementary Material 2.

Supplementary Material 3.

Supplementary Material 4.

Supplementary Material 5.

Supplementary Material 6.

Supplementary Material 7.

Supplementary Material 8.

## Data Availability

All data generated or during this study are included in this published article and the supplementary materials. GWAS summary statistics for periodontal disease and IMID were publicly available at R9 GWAS data which published by the FinnGen Consortium (https://r9.finngen.fi/), and GWAS round 2 data was published by the UKB (http://www.nealelab.is/uk-biobank). In addition, the GLIDE consortium contributed clinically diagnosed periodontitis GWAS data, refer to 10.1038/s41467-019-10630-1.

## Abbreviations

IMID: Immune-mediated inflammatory disorders
MR: Mendelian randomization
GWAS: Genome-wide association studies
UKB: UK Biobank
GLIDE: Gene Lifestyle Interactions in Dental Endpoints
SLE: Systemic lupus erythematosus
IBD: Inflammatory bowel disease
UC: Ulcerative colitis
SNPs: Single nucleotide polymorphisms
RCTs: Randomized controlled trials
LD: Linkage disequilibrium
WM: Weighted median
IVW: Inverse variance weighted
OR: Odds ratios
SD: Standard deviation
