# Supplementary material for: Immune-mediated inflammatory diseases and periodontal disease: a bidirectional two-sample mendelian randomization study
Source: BMC Immunol. 2024 Jun 28;25:39. doi: 10.1186/s12865-024-00634-y (PMC11212394; doi:10.1186/s12865-024-00634-y)
Supplement: Supplementary file 1 — Supplementary Material 1. [file 12865_2024_634_MOESM1_ESM.docx]

**Table S1**  **Cases and controls from the FinnGen Consortium and UKB for ten kinds of IMID**

| **Database** | **Cases/**  **controls** | ***Hyperthyroidism*** | ***Hypothyroidism*** | ***SLE*** | ***Crohn’s disease (small intestine)*** | ***Crohn’s disease (large intestine)*** | ***IBD*** | ***UC*** | ***Psoriasis*** | ***Rheumatoid*** ***arthritis*** | ***Sjogren syndrome*** |
| --- | --- | --- | --- | --- | --- | --- | --- | --- | --- | --- | --- |
| **FinnGen** | **Cases** | 1828 | 36321 | 1023 | 2004 | 1581 | 7625 | 5034 | 9267 | 12555 | 2495 |
|  | **controls** | 279855 | 274069 | 281127 | 359927 | 359927 | 359927 | 371530 | 364071 | 240862 | 365533 |
| **UKB** | **Cases** | 2730 | 17574 | 415 | 249 | 336 | 150 | 1916 | 4192 | 4017 | 338 |
|  | **controls** | 358411 | 343567 | 360726 | 360945 | 360858 | 360991 | 359225 | 356949 | 357124 | 360803 |

Note: UKB, UK Biobank; IMID, immune-mediated inflammatory disorders; SLE, Systemic lupus erythematosus; IBD, Inflammatory bowel disease; UC, Ulcerative colitis.
