# Supplementary material for: Immune-mediated inflammatory diseases and periodontal disease: a bidirectional two-sample mendelian randomization study
Source: BMC Immunol. 2024 Jun 28;25:39. doi: 10.1186/s12865-024-00634-y (PMC11212394; doi:10.1186/s12865-024-00634-y)
Supplement: Supplementary file 4 — Supplementary Material 4. [file 12865_2024_634_MOESM4_ESM.docx]

**Table S4 MR analysis for different databases and methods of IMIDs to periodontal disease**

| **Database** | **Exposure** | **Methods** | **OR** | **CI up** | **CI low** | **P-value** |
| --- | --- | --- | --- | --- | --- | --- |
| **FinnGen to GLIDE** | ***hyperthyroidism*** | MR Egger | 1.067 | 0.955 | 1.192 | 0.283 |
|  |  | Weighted median | 0.971 | 0.912 | 1.034 | 0.359 |
|  |  | Inverse variance weighted | 0.977 | 0.935 | 1.020 | 0.280 |
|  | ***hypothyroidism*** | MR Egger | 0.997 | 0.899 | 1.105 | 0.948 |
|  |  | Weighted median | 0.971 | 0.896 | 1.053 | 0.481 |
|  |  | Inverse variance weighted | 0.990 | 0.946 | 1.036 | 0.650 |
|  | ***SLE*** | MR Egger | 1.000 | 0.896 | 1.116 | 0.994 |
|  |  | Weighted median | 1.067 | 1.010 | 1.127 | 0.021 |
|  |  | Inverse variance weighted | 1.079 | 1.032 | 1.128 | <0.001 |
|  | ***Crohn’s disease (small intestine)*** | MR Egger | 0.951 | 0.719 | 1.258 | 0.736 |
|  |  | Weighted median | 1.009 | 0.929 | 1.096 | 0.836 |
|  |  | Inverse variance weighted | 1.020 | 0.939 | 1.109 | 0.633 |
|  | ***Crohn’s disease (large intestine)*** | MR Egger | 0.951 | 0.674 | 1.341 | 0.821 |
|  |  | Weighted median | 0.962 | 0.870 | 1.063 | 0.449 |
|  |  | Inverse variance weighted | 0.968 | 0.889 | 1.054 | 0.457 |
|  | ***IBD*** | MR Egger | 0.898 | 0.795 | 1.013 | 0.089 |
|  |  | Weighted median | 0.957 | 0.900 | 1.018 | 0.164 |
|  |  | Inverse variance weighted | 0.984 | 0.942 | 1.028 | 0.473 |
|  | ***UC*** | MR Egger | 0.917 | 0.795 | 1.058 | 0.244 |
|  |  | Weighted median | 0.969 | 0.917 | 1.023 | 0.255 |
|  |  | Inverse variance weighted | 0.981 | 0.943 | 1.022 | 0.358 |
|  | ***Psoriasis*** | MR Egger | 0.956 | 0.792 | 1.155 | 0.648 |
|  |  | Weighted median | 1.027 | 0.941 | 1.121 | 0.554 |
|  |  | Inverse variance weighted | 1.015 | 0.953 | 1.080 | 0.644 |
|  | ***Rheumatoid arthritis*** | MR Egger | 1.013 | 0.876 | 1.171 | 0.866 |
|  |  | Weighted median | 1.037 | 0.944 | 1.138 | 0.450 |
|  |  | Inverse variance weighted | 1.028 | 0.953 | 1.109 | 0.472 |
|  | ***Sjogren syndrome*** | MR Egger | 0.985 | 0.879 | 1.104 | 0.808 |
|  |  | Weighted median | 1.072 | 1.003 | 1.145 | 0.041 |
|  |  | Inverse variance weighted | 1.082 | 1.012 | 1.157 | 0.022 |
| **UKB to FinnGen** | ***hyperthyroidism*** | MR Egger | 1.76*10^2^ | 1.04*10^-22^ | 2.95*10^26^ | 0.886 |
|  |  | Weighted median | 1.78 | 3.45*10^-2^ | 9.23*10 | 0.774 |
|  |  | Inverse variance weighted | 6.40 | 6.34*10^-2^ | 6.45*10^2^ | 0.430 |
|  | ***hypothyroidism*** | MR Egger | 1.34 | 7.24*10^-1^ | 2.47 | 0.357 |
|  |  | Weighted median | 1.37 | 8.69*10^-1^ | 2.18 | 0.174 |
|  |  | Inverse variance weighted | 1.52 | 1.13 | 2.04 | 0.005 |
|  | ***SLE*** | MR Egger | 8.29*10 | 1.40*10^-2^ | 4.91*10^5^ | 0.424 |
|  |  | Weighted median | 6.79*10 | 1.50*10^-2^ | 3.07*10^5^ | 0.326 |
|  |  | Inverse variance weighted | 7.57 | 5.70*10^-3^ | 1.00*10^4^ | 0.581 |
|  | ***Crohn’s disease (small intestine)*** | MR Egger | 5.63 | 8.19*10^-6^ | 3.88*10^6^ | 0.811 |
|  |  | Weighted median | 2.61*10^-1^ | 8.48*10^-4^ | 8.03*10 | 0.646 |
|  |  | Inverse variance weighted | 9.58*10^-2^ | 6.80*10^-4^ | 1.35*10 | 0.353 |
|  | ***Crohn’s disease (large intestine)*** | MR Egger | 4.76 | 1.36*10^-8^ | 1.67*10^9^ | 0.891 |
|  |  | Weighted median | 1.09 | 1.42*10^-4^ | 8.40*10^3^ | 0.984 |
|  |  | Inverse variance weighted | 2.90*10^-1^ | 7.04*10^-5^ | 1.19*10^3^ | 0.771 |
|  | ***IBD*** | MR Egger | 3.97*10^-1^ | 1.86*10^-2^ | 8.48 | 0.586 |
|  |  | Weighted median | 3.27*10^-1^ | 3.63*10^-2^ | 2.95 | 0.320 |
|  |  | Inverse variance weighted | 3.38*10^-1^ | 4.96*10^-2^ | 2.31 | 0.268 |
|  | ***UC*** | MR Egger | 1.81*10^-3^ | 8.43*10^-10^ | 3.87*10^3^ | 0.444 |
|  |  | Weighted median | 2.74*10^-1^ | 8.15*10^-4^ | 9.25*10 | 0.663 |
|  |  | Inverse variance weighted | 1.70 | 1.39*10^-2^ | 2.09*10^2^ | 0.828 |
|  | ***Psoriasis*** | MR Egger | 1.31 | 1.21*10^-1^ | 1.42*10 | 0.829 |
|  |  | Weighted median | 5.02 | 8.38*10^-1^ | 3.00*10 | 0.077 |
|  |  | Inverse variance weighted | 1.26 | 2.11*10^-1^ | 7.55 | 0.799 |
|  | ***Rheumatoid arthritis*** | MR Egger | 4.01 | 1.74*10^-10^ | 9.26*10^10^ | 0.920 |
|  |  | Weighted median | 7.95*10^-2^ | 7.78*10^-4^ | 8.13 | 0.284 |
|  |  | Inverse variance weighted | 7.06*10^-1^ | 2.19*10^-3^ | 2.28*10^2^ | 0.906 |
| **UKB to GLIDE** | ***hyperthyroidism*** | MR Egger | 3.22*10 | 2.57*10^-32^ | 4.04*10^34^ | 0.934 |
|  |  | Weighted median | 1.19*10^-4^ | 4.31*10^-9^ | 3.28 | 0.083 |
|  |  | Inverse variance weighted | 3.21*10^-4^ | 5.80*10^-8^ | 1.78 | 0.067 |
|  | ***hypothyroidism*** | MR Egger | 7.50*10^-1^ | 1.01*10^-1^ | 5.56 | 0.779 |
|  |  | Weighted median | 4.52*10^-1^ | 1.11*10^-1^ | 1.85 | 0.269 |
|  |  | Inverse variance weighted | 8.91*10^-1^ | 3.59*10^-1^ | 2.21 | 0.803 |
|  | ***SLE*** | MR Egger | 5.13*10^-3^ | 2.11*10^-11^ | 1.25*10^6^ | 0.646 |
|  |  | Weighted median | 1.91 | 3.22*10^-7^ | 1.13*10^7^ | 0.935 |
|  |  | Inverse variance weighted | 1.69 | 1.86*10^-8^ | 1.53*10^8^ | 0.955 |
|  | ***Crohn’s disease (small intestine)*** | MR Egger | 2.33*10^3^ | 9.49*10^-34^ | 5.71*10^39^ | 0.865 |
|  |  | Weighted median | 6.68*10 | 5.52*10^-15^ | 8.10*10^17^ | 0.824 |
|  |  | Inverse variance weighted | 1.58*10 | 1.07*10^-15^ | 2.33*10^17^ | 0.885 |
|  | ***Crohn’s disease (large intestine)*** | MR Egger | 9.88*10^-4^ | 8.14*10^-38^ | 1.20*10^31^ | 0.879 |
|  |  | Weighted median | 1.15*10^-1^ | 2.68*10^-11^ | 4.96*10^8^ | 0.849 |
|  |  | Inverse variance weighted | 3.32*10^-2^ | 9.56*10^-13^ | 1.16*10^9^ | 0.783 |
|  | ***IBD*** | MR Egger | 6.41*10^-3^ | 9.45*10^-11^ | 4.35*10^5^ | 0.621 |
|  |  | Weighted median | 7.47*10^-3^ | 5.09*10^-10^ | 1.10*10^5^ | 0.561 |
|  |  | Inverse variance weighted | 4.75*10^-3^ | 1.72*10^-9^ | 1.31*10^4^ | 0.480 |
|  | ***UC*** | MR Egger | 7.84*10^-20^ | 5.74*10^-52^ | 1.07*10^13^ | 0.288 |
|  |  | Weighted median | 1.71*10^-2^ | 2.89*10^-10^ | 1.01*10^6^ | 0.656 |
|  |  | Inverse variance weighted | 6.48*10^-5^ | 3.93*10^-11^ | 1.07*10^2^ | 0.187 |
|  | ***Psoriasis*** | MR Egger | 6.67 | 2.20*10^-2^ | 2.02*10^3^ | 0.530 |
|  |  | Weighted median | 9.24*10^-1^ | 9.06*10^-3^ | 9.43*10 | 0.973 |
|  |  | Inverse variance weighted | 1.57 | 2.01*10^-2^ | 1.23*10^2^ | 0.839 |
|  | ***Rheumatoid arthritis*** | MR Egger | 5.51 | 2.61*10^-3^ | 1.16*10^4^ | 0.692 |
|  |  | Weighted median | 2.80*10^-1^ | 2.46*10^-3^ | 3.18*10 | 0.598 |
|  |  | Inverse variance weighted | 1.68*10^-1^ | 1.61*10^-3^ | 1.75*10 | 0.452 |

**Note**: MR, Mendelian randomization; IMID, immune-mediated inflammatory disorders; OR, Odds ratios; CI, confidence interval; UKB, UK Biobank; GLIDE, Gene Lifestyle Interactions in Dental Endpoints; SLE, Systemic lupus erythematosus; IBD, Inflammatory bowel disease; UC, Ulcerative colitis.
