# Supplementary material for: Immune-mediated inflammatory diseases and periodontal disease: a bidirectional two-sample mendelian randomization study
Source: BMC Immunol. 2024 Jun 28;25:39. doi: 10.1186/s12865-024-00634-y (PMC11212394; doi:10.1186/s12865-024-00634-y)
Supplement: Supplementary file 5 — Supplementary Material 5. [file 12865_2024_634_MOESM5_ESM.docx]

**Table S5** **MR analysis for different databases and methods of periodontal disease to IMIDs**

| **Database** | **Outcome** | **Methods** | **OR** | **CI up** | **CI low** | **P-value** |
| --- | --- | --- | --- | --- | --- | --- |
| **FinnGen to UKB** | ***hyperthyroidism*** | MR Egger | 0.9995 | 0.9959 | 1.0031 | 0.778 |
|  |  | Weighted median | 0.9993 | 0.9959 | 1.0027 | 0.688 |
|  |  | Inverse variance weighted | 0.9991 | 0.9966 | 1.0016 | 0.467 |
|  | ***hypothyroidism*** | MR Egger | 0.9983 | 0.9873 | 1.0093 | 0.762 |
|  |  | Weighted median | 1.0019 | 0.9930 | 1.0109 | 0.676 |
|  |  | Inverse variance weighted | 1.0015 | 0.9940 | 1.0091 | 0.694 |
|  | ***SLE*** | MR Egger | 1.0000 | 0.9985 | 1.0014 | 0.946 |
|  |  | Weighted median | 1.0000 | 0.9987 | 1.0013 | 0.964 |
|  |  | Inverse variance weighted | 0.9997 | 0.9987 | 1.0006 | 0.508 |
|  | ***Crohn’s disease (small intestine)*** | MR Egger | 1.0009 | 0.9998 | 1.0021 | 0.137 |
|  |  | Weighted median | 1.0002 | 0.9992 | 1.0013 | 0.663 |
|  |  | Inverse variance weighted | 1.0003 | 0.9995 | 1.0011 | 0.477 |
|  | ***Crohn’s disease (large intestine)*** | MR Egger | 1.0006 | 0.9994 | 1.0019 | 0.355 |
|  |  | Weighted median | 1.0004 | 0.9992 | 1.0015 | 0.535 |
|  |  | Inverse variance weighted | 1.0004 | 0.9995 | 1.0013 | 0.354 |
|  | ***IBD*** | MR Egger | 1.0000 | 0.9991 | 1.0009 | 0.977 |
|  |  | Weighted median | 1.0001 | 0.9993 | 1.0010 | 0.738 |
|  |  | Inverse variance weighted | 1.0004 | 0.9997 | 1.0010 | 0.266 |
|  | ***UC*** | MR Egger | 1.0008 | 0.9969 | 1.0047 | 0.693 |
|  |  | Weighted median | 1.0013 | 0.9980 | 1.0046 | 0.440 |
|  |  | Inverse variance weighted | 1.0010 | 0.9984 | 1.0036 | 0.449 |
|  | ***Psoriasis*** | MR Egger | 1.0022 | 0.9977 | 1.0067 | 0.359 |
|  |  | Weighted median | 1.0038 | 0.9994 | 1.0082 | 0.088 |
|  |  | Inverse variance weighted | 1.0024 | 0.9993 | 1.0054 | 0.133 |
|  | ***Rheumatoid arthritis*** | MR Egger | 1.0046 | 0.9996 | 1.0097 | 0.092 |
|  |  | Weighted median | 1.0018 | 0.9977 | 1.0060 | 0.384 |
|  |  | Inverse variance weighted | 1.0016 | 0.9979 | 1.0053 | 0.392 |
|  | ***Sjogren syndrome*** | MR Egger | 0.9989 | 0.9977 | 1.0002 | 0.114 |
|  |  | Weighted median | 0.9991 | 0.9980 | 1.0003 | 0.146 |
|  |  | Inverse variance weighted | 0.9992 | 0.9983 | 1.0001 | 0.067 |
| **GLIDE to FinnGen** | ***hyperthyroidism*** | MR Egger | 1.0171 | 0.8525 | 1.2134 | 0.860 |
|  |  | Weighted median | 0.9728 | 0.8218 | 1.1515 | 0.748 |
|  |  | Inverse variance weighted | 0.9563 | 0.8219 | 1.1126 | 0.563 |
|  | ***hypothyroidism*** | MR Egger | 1.0182 | 0.9661 | 1.0731 | 0.538 |
|  |  | Weighted median | 1.0193 | 0.9756 | 1.0648 | 0.393 |
|  |  | Inverse variance weighted | 1.0173 | 0.9771 | 1.0592 | 0.404 |
|  | ***SLE*** | MR Egger | 0.7636 | 0.6222 | 0.9370 | 0.061 |
|  |  | Weighted median | 0.8072 | 0.6452 | 1.0097 | 0.061 |
|  |  | Inverse variance weighted | 0.8079 | 0.6764 | 0.9650 | 0.019 |
|  | ***Crohn’s disease (small intestine)*** | MR Egger | 0.9455 | 0.7987 | 1.1193 | 0.551 |
|  |  | Weighted median | 0.9433 | 0.7888 | 1.1280 | 0.522 |
|  |  | Inverse variance weighted | 0.9422 | 0.8152 | 1.0890 | 0.420 |
|  | ***Crohn’s disease (large intestine)*** | MR Egger | 1.1183 | 0.9241 | 1.3532 | 0.315 |
|  |  | Weighted median | 1.0734 | 0.8881 | 1.2974 | 0.464 |
|  |  | Inverse variance weighted | 1.0607 | 0.9007 | 1.2492 | 0.480 |
|  | ***IBD*** | MR Egger | 1.0465 | 0.9524 | 1.1499 | 0.398 |
|  |  | Weighted median | 1.0388 | 0.9478 | 1.1386 | 0.416 |
|  |  | Inverse variance weighted | 1.0288 | 0.9531 | 1.1106 | 0.466 |
|  | ***UC*** | MR Egger | 1.1108 | 0.9974 | 1.2370 | 0.128 |
|  |  | Weighted median | 1.1103 | 0.9913 | 1.2436 | 0.071 |
|  |  | Inverse variance weighted | 1.0687 | 0.9744 | 1.1720 | 0.158 |
|  | ***Psoriasis*** | MR Egger | 0.9956 | 0.9199 | 1.0776 | 0.918 |
|  |  | Weighted median | 1.0052 | 0.9295 | 1.0871 | 0.896 |
|  |  | Inverse variance weighted | 1.0065 | 0.9404 | 1.0771 | 0.853 |
|  | ***Rheumatoid arthritis*** | MR Egger | 0.9450 | 0.8526 | 1.0473 | 0.341 |
|  |  | Weighted median | 0.9662 | 0.9004 | 1.0368 | 0.339 |
|  |  | Inverse variance weighted | 0.9555 | 0.8816 | 1.0356 | 0.268 |
|  | ***Sjogren syndrome*** | MR Egger | 0.9646 | 0.7963 | 1.1685 | 0.731 |
|  |  | Weighted median | 0.9959 | 0.8579 | 1.1561 | 0.957 |
|  |  | Inverse variance weighted | 0.9774 | 0.8428 | 1.1336 | 0.763 |
| **UKB to FinnGen** | ***hyperthyroidism*** | MR Egger | 7.06*10^-8^ | 1.04*10^-17^ | 4.81*10^2^ | 0.164 |
|  |  | Weighted median | 1.92*10^-2^ | 8.96*10^-12^ | 4.12*10^7^ | 0.718 |
|  |  | Inverse variance weighted | 5.59*10^-9^ | 1.43*10^-15^ | 2.18*10^-2^ | 0.014 |
|  | ***hypothyroidism*** | MR Egger | 1.15 | 6.00*10^-3^ | 2.19*10^2^ | 0.960 |
|  |  | Weighted median | 5.54 | 3.98*10^-2^ | 7.69*10^2^ | 0.497 |
|  |  | Inverse variance weighted | 3.98 | 1.16*10^-1^ | 1.37*10^2^ | 0.444 |
|  | ***SLE*** | MR Egger | 1.32*10^-3^ | 9.74*10^-17^ | 1.80*10^10^ | 0.671 |
|  |  | Weighted median | 1.95*10^-4^ | 1.40*10^-17^ | 2.73*10^9^ | 0.580 |
|  |  | Inverse variance weighted | 3.47*10^-5^ | 4.88*10^-14^ | 2.47*10^4^ | 0.323 |
|  | ***Crohn’s disease (small intestine)*** | MR Egger | 5.12*10^4^ | 7.25*10^-6^ | 3.61*10^14^ | 0.356 |
|  |  | Weighted median | 6.20*10^4^ | 1.02*10^-5^ | 3.78*10^14^ | 0.337 |
|  |  | Inverse variance weighted | 5.12*10^3^ | 1.48*10^-3^ | 1.78*10^10^ | 0.266 |
|  | ***Crohn’s disease (large intestine)*** | MR Egger | 1.55*10^7^ | 5.62*10^-5^ | 4.28*10^18^ | 0.227 |
|  |  | Weighted median | 4.72*10^5^ | 1.75*10^-5^ | 1.27*10^16^ | 0.286 |
|  |  | Inverse variance weighted | 7.93*10^6^ | 2.10*10^-1^ | 2.99*10^14^ | 0.074 |
|  | ***IBD*** | MR Egger | 1.17*10^-2^ | 9.70*10^-9^ | 1.41*10^4^ | 0.538 |
|  |  | Weighted median | 3.96*10 | 1.46*10^-4^ | 1.08*10^7^ | 0.564 |
|  |  | Inverse variance weighted | 2.43*10^-2^ | 2.26*10^-6^ | 2.61*10^2^ | 0.432 |
|  | ***UC*** | MR Egger | 3.36*10^-3^ | 4.18*10^-9^ | 2.70*10^3^ | 0.418 |
|  |  | Weighted median | 8.82*10^-1^ | 6.08*10^-7^ | 1.28*10^6^ | 0.986 |
|  |  | Inverse variance weighted | 1.77*10^-3^ | 1.92*10^-7^ | 1.62*10 | 0.173 |
|  | ***Psoriasis*** | MR Egger | 1.55*10^-2^ | 6.54*10^-7^ | 3.67*10^2^ | 0.424 |
|  |  | Weighted median | 3.10*10^-4^ | 8.05*10^-9^ | 1.20*10 | 0.134 |
|  |  | Inverse variance weighted | 2.71*10^-1^ | 3.07*10^-4^ | 2.40*10^2^ | 0.706 |
|  | ***Rheumatoid arthritis*** | MR Egger | 4.61*10^-3^ | 4.36*10^-7^ | 4.88*10 | 0.264 |
|  |  | Weighted median | 2.30*10^-3^ | 5.15*10^-7^ | 1.02*10 | 0.156 |
|  |  | Inverse variance weighted | 5.72*10^-1^ | 1.02*10^-3^ | 3.22*10^2^ | 0.863 |
|  | ***Sjogren syndrome*** | MR Egger | 5.54*10 | 2.50*10^-7^ | 1.23*10^10^ | 0.685 |
|  |  | Weighted median | 1.04 | 2.48*10^-9^ | 4.40*10^8^ | 0.997 |
|  |  | Inverse variance weighted | 2.10*10^-2^ | 5.05*10^-8^ | 8.76*10^3^ | 0.559 |

**Note**: MR, Mendelian randomization; IMID, immune-mediated inflammatory disorders; OR, Odds ratios; CI, confidence interval; UKB, UK Biobank; GLIDE, Gene Lifestyle Interactions in Dental Endpoints; SLE, Systemic lupus erythematosus; IBD, Inflammatory bowel disease; UC, Ulcerative colitis.
