# Supplementary material for: Immune-mediated inflammatory diseases and periodontal disease: a bidirectional two-sample mendelian randomization study
Source: BMC Immunol. 2024 Jun 28;25:39. doi: 10.1186/s12865-024-00634-y (PMC11212394; doi:10.1186/s12865-024-00634-y)
Supplement: Supplementary file 6 — Supplementary Material 6. [file 12865_2024_634_MOESM6_ESM.pdf]

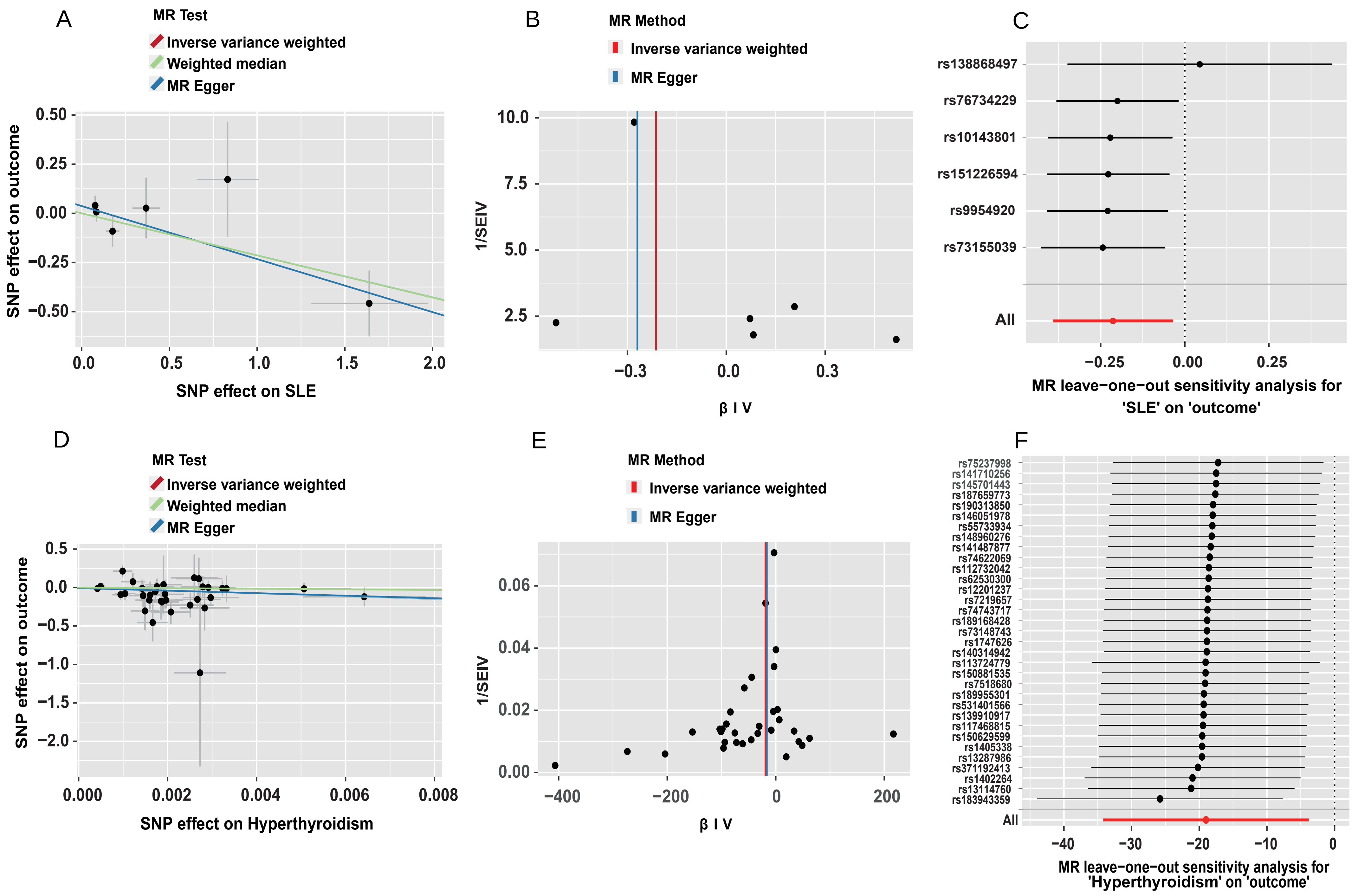
A
MR Test
Inverse variance weighted
Weighted median
MR Egger
SNP effect on outcome
SNP effect on SLE
B
MR Method
Inverse variance weighted
MR Egger
1/SEIV
β I V
C
rs138868497
rs76734229
rs10143801
rs151226594
rs9954920
rs73155039
All
MR leave−one−out sensitivity analysis for
'SLE' on 'outcome'
D
MR Test
Inverse variance weighted
Weighted median
MR Egger
SNP effect on outcome
SNP effect on Hyperthyroidism
E
MR Method
Inverse variance weighted
MR Egger
1/SEIV
β I V
F
rs75237998
rs141710256
rs145701443
rs187659773
rs190313850
rs146051978
rs55733934
rs148960276
rs141487877
rs74622069
rs112732042
rs62530300
rs12201237
rs7219657
rs74743717
rs189168428
rs73148743
rs1747626
rs140314942
rs113724779
rs150881535
rs7518680
rs189955301
rs531401566
rs139910917
rs117468815
rs150629599
rs1405338
rs13287986
rs371192413
rs1402264
rs13114760
rs183943359
All
MR leave−one−out sensitivity analysis for
'Hyperthyroidism' on 'outcome'

Figure S1 Three kinds of plots for the causal relationship between IMID and periodontal disease. When causal effects of periodontal disease (GLIDE) to IMID (FinnGen); A, scatter plot of SLE; B, funnel plot of SLE; C, leave-one-out plot of SLE. When causal effects of periodontal disease (UKB) to IMID (FinnGen); D, scatter plot of hyperthyroidism; E, funnel plot of hyperthyroidism; F, leave-one-out plot of hyperthyroidism. IMID, Immune-mediated inflammatory disorders; MR, Mendelian randomization; SNP, Single nucleotide polymorphism; SLE, Systemic lupus erythematosus.
