# Supplementary material for: Immune-mediated inflammatory diseases and periodontal disease: a bidirectional two-sample mendelian randomization study
Source: BMC Immunol. 2024 Jun 28;25:39. doi: 10.1186/s12865-024-00634-y (PMC11212394; doi:10.1186/s12865-024-00634-y)
Supplement: Supplementary file 7 — Supplementary Material 7. [file 12865_2024_634_MOESM7_ESM.pdf]

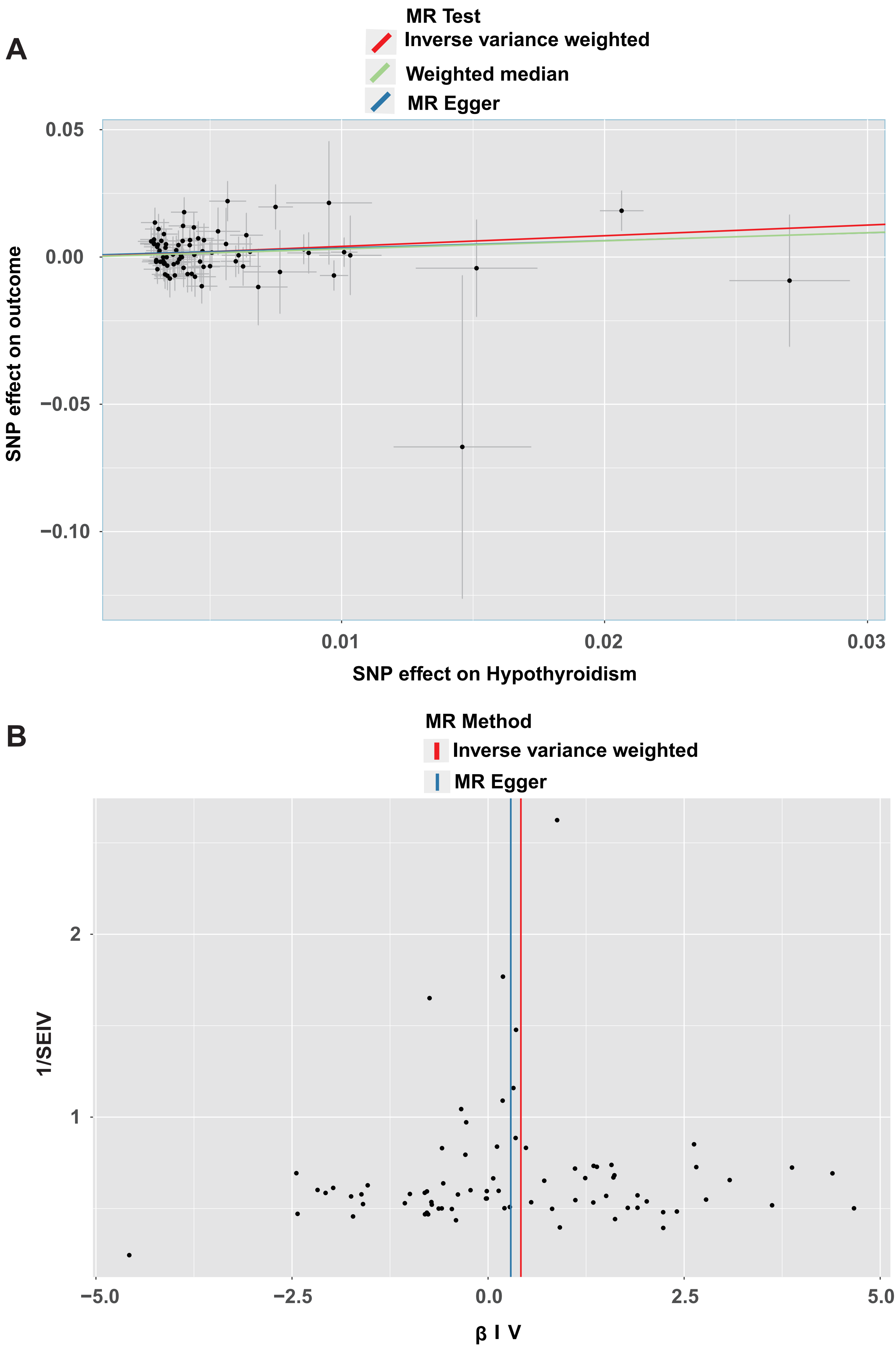
A
MR Test
Inverse variance weighted
Weighted median
MR Egger
0.05
0.00
−0.05
−0.10
SNP effect on outcome
0.01
0.02
0.03
SNP effect on Hypothyroidism
B
MR Method
Inverse variance weighted
MR Egger
2
1
1/SEIV
−5.0
−2.5
0.0
2.5
5.0
β I V

Figure S2 Scatter plot and funnel plot of hypothyroidism. The association between IMIDs (UKB) and periodontal disease (FinnGen), A, scatter plot of hypothyroidism; B, funnel plot of hypothyroidism. MR, Mendelian randomization.
