# Supplementary material for: Immune-mediated inflammatory diseases and periodontal disease: a bidirectional two-sample mendelian randomization study
Source: BMC Immunol. 2024 Jun 28;25:39. doi: 10.1186/s12865-024-00634-y (PMC11212394; doi:10.1186/s12865-024-00634-y)
Supplement: Supplementary file 8 — Supplementary Material 8. [file 12865_2024_634_MOESM8_ESM.pdf]

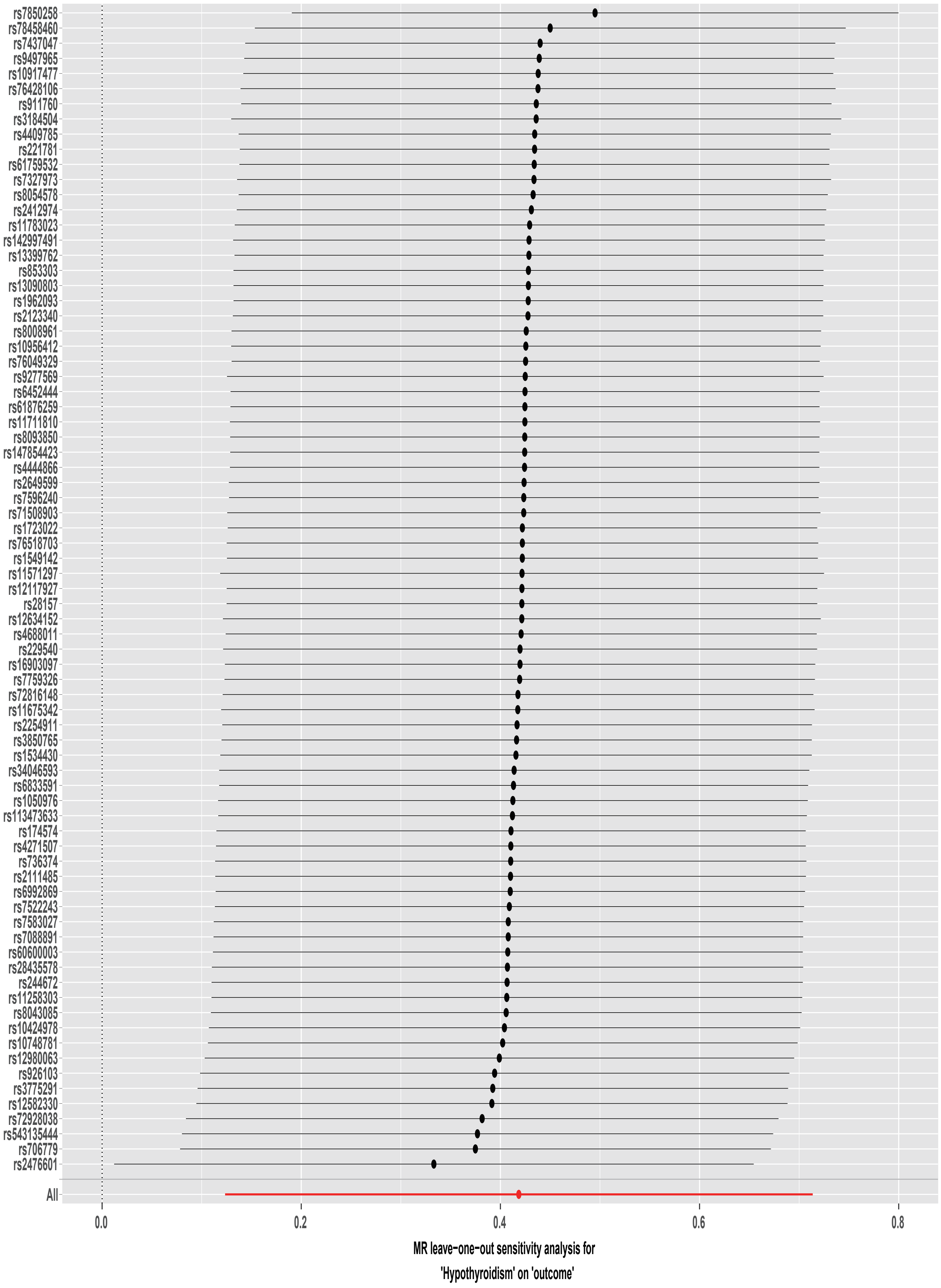

rs7850258
rs78458460
rs7437047
rs9497965
rs10917477
rs76428106
rs911760
rs3184504
rs4409785
rs221781
rs61759532
rs7327973
rs8054578
rs2412974
rs11783023
rs142997491
rs13399762
rs853303
rs13090803
rs1962093
rs2123340
rs8008961
rs10956412
rs76049329
rs9277569
rs6452444
rs61876259
rs11711810
rs8093850
rs147854423
rs4444866
rs2649599
rs7596240
rs71508903
rs1723022
rs76518703
rs1549142
rs11571297
rs12117927
rs28157
rs12634152
rs4688011
rs229540
rs16903097
rs7759326
rs72816148
rs11675342
rs2254911
rs3850765
rs1534430
rs34046593
rs6833591
rs1050976
rs113473633
rs174574
rs4271507
rs736374
rs2111485
rs6992869
rs7522243
rs7583027
rs7088891
rs60600003
rs28435578
rs244672
rs11258303
rs8043085
rs10424978
rs10748781
rs12980063
rs926103
rs3775291
rs12582330
rs72928038
rs543135444
rs706779
rs2476601
All
0.0
0.2
0.4
0.6
0.8
MR leave−one−out sensitivity analysis for
'Hypothyroidism' on 'outcome'

Figure S3 Leave-one-out plot of hypothyroidism. The association between IMIDs (UKB) and periodontal disease (FinnGen). IMID, Immune-mediated inflammatory disorders; MR, Mendelian randomization; SNP, Single nucleotide polymorphism.
